# Authorship Bias in Violence Risk Assessment? A Systematic Review and Meta-Analysis

**DOI:** 10.1371/journal.pone.0072484

**Published:** 2013-09-02

**Authors:** Jay P. Singh, Martin Grann, Seena Fazel

**Affiliations:** 1 Psychiatric/Psychological Service, Department of Justice, Zürich, Switzerland; 2 Faculty of Health Sciences, Molde University College, Molde, Norway; 3 Centre for Violence Prevention, Department of Medical Epidemiology and Biostatistics, Karolinska Institute, Stockholm, Sweden; 4 Department of Psychiatry, University of Oxford, Oxford, United Kingdom; University of Illinois-Chicago, United States of America

## Abstract

Various financial and non-financial conflicts of interests have been shown to influence the reporting of research findings, particularly in clinical medicine. In this study, we examine whether this extends to prognostic instruments designed to assess violence risk. Such instruments have increasingly become a routine part of clinical practice in mental health and criminal justice settings. The present meta-analysis investigated whether an authorship effect exists in the violence risk assessment literature by comparing predictive accuracy outcomes in studies where the individuals who designed these instruments were study authors with independent investigations. A systematic search from 1966 to 2011 was conducted using PsycINFO, EMBASE, MEDLINE, and US National Criminal Justice Reference Service Abstracts to identify predictive validity studies for the nine most commonly used risk assessment tools. Tabular data from 83 studies comprising 104 samples was collected, information on two-thirds of which was received directly from study authors for the review. Random effects subgroup analysis and metaregression were used to explore evidence of an authorship effect. We found a substantial and statistically significant authorship effect. Overall, studies authored by tool designers reported predictive validity findings around two times higher those of investigations reported by independent authors (DOR = 6.22 [95% CI = 4.68–8.26] in designers' studies vs. DOR = 3.08 [95% CI = 2.45–3.88] in independent studies). As there was evidence of an authorship effect, we also examined disclosure rates. None of the 25 studies where tool designers or translators were also study authors published a conflict of interest statement to that effect, despite a number of journals requiring that potential conflicts be disclosed. The field of risk assessment would benefit from routine disclosure and registration of research studies. The extent to which similar conflict of interests exists in those developing risk assessment guidelines and providing expert testimony needs clarification.

## Introduction

A variety of financial and non-financial conflict of interests have been identified in medical and behavioral research, resulting in calls for more transparent reporting of potential conflicts, efforts to register all research activity in certain fields, and careful examination of sources of heterogeneity in meta-analytic investigations. To date, much of the research in this area has focused on clinical trials. There is consistent and robust evidence that industry-sponsored trials are more likely to report positive significant findings [1], [2], with independent replications of some research having discovered inflated effects. Little work has been done for study designs other than clinical trials, but reviews suggest clear design-related biases in studies of diagnostic and prognostic tools [3]. The importance of investigating the presence of such biases is clear–the credibility of research findings may be questioned in the absence of disclosures.

In the fields of psychiatry and psychology, there has been an increasing use of violence risk assessment tools over the past three decades [4]. The demand for such tools has increased with the rising call for the use of evidence-based, structured, and transparent decision-making processes that may result in deprivation of individual liberty, or in permitting leave or release in detainees. In addition, the increased use of violence risk assessment tools has been fuelled by a number of high-profile cases in recent years, such as homicides by psychiatric patients, attempted terrorist attacks, and school shootings.

Thus, these tools have been developed as structured methods of assessing the risk of violence posed by forensic psychiatric patients and other high risk groups such as prisoners and probationers. Contemporary risk assessment tools largely follow either the actuarial or structured clinical judgment (SCJ) approach. The actuarial approach involves scoring patients on a predetermined set of weighted risk and protective factors found to be statistically associated with the antisocial outcome of interest. Patients' total scores are algorithmically cross-referenced with manualized tables in order to produce a probabilistic estimate of risk.

SCJ assessments involve administrators examining the presence or absence of theoretically, clinically, and/or empirically supported risk and protective factors. This information is then used to develop a risk formulation based on the clinician's experience and intuition. As part of this formulation, examinees are assigned to one of three risk categories: low, moderate, or high. The proliferation of research into the predictive validity of both actuarial and SCJ tools [5] has largely been driven by influential reports that unstructured clinical predictions are not accurate [6].

A conflict of interest may result when the designers of a risk assessment tool investigate the predictive validity of the very same instrument in validation studies. Tool designers may have a vested interest in their measure performing well, as such empirical support can lead to both financial benefits (e.g., selling tool manuals and coding sheets, offering training sessions, being hired as an expert witness, attracting funding) as well as non-financial benefits (e.g., increased recognition in the field and more opportunities for career advancement). This may result in what we have called an *authorship effect* whereby the designers of a risk assessment tool find more positive significant results when investigating their own tool's predictive validity than do independent researchers.

The majority of the most commonly used risk assessment tools were developed in English and these have all been translated into a great number of other languages. In most cases, researchers and experts who have translated the tool have received formal permission from the designers to do so and, as a consequence, exert a more or less formal or informal ownership of the tool in their home country or region. Similar to the case of the designers, it is possible that translators might also have a conflict of interest that manifests in a form of bias.

### Previous Research on the Authorship Effect

The meta-analytic evidence concerning the existence of an authorship effect in the risk assessment literature is limited and reports contrasting conclusions [7]–[9]. First, Blair and colleagues [7] explored an authorship effect using the literature on the Violence Risk Appraisal Guide (VRAG) [10], [11], the Sex Offender Risk Appraisal Guide (SORAG) [10], [11], and the Static-99 [12], [13]–actuarial risk assessment tools designed for use with adult offenders. Evidence of an authorship effect was found in that studies on which a tool author was also a study author (*r* = 0.37; 95% CI = 0.33–0.41) produced higher rates of predictive validity than studies conducted by independent researchers (*r* = 0.28; 95% CI = 0.26–0.31). This meta-analysis was limited as only published studies were included and studies with overlapping samples were not excluded.

Second, Harris, Rice, and Quinsey [8], co-authors of two of the instruments in the previous review (VRAG and SORAG), re-analyzed the predictive validity literatures of their instruments including unpublished studies and avoiding overlapping samples. Using a different outcome measure – the area under the receiver operating characteristic curve (AUC) – the review found that studies in which a tool author was also a study author produced similar effect estimates to studies conducted by independent investigators. However, the authors provided no statistical tests to support their conclusions and the range of instruments included remained very limited. This review also did not investigate the evidence for an authorship effect in the published and unpublished literature, separately. Finally, methodologists have recently suggested that the AUC may not be able to differentiate between models that discriminate better than chance [14]–[16], suggesting that these findings should be interpreted with caution.

Finally, Guy [9], as part of a Master's thesis supervised by designers of a set of well-known SCJ tools, investigated whether being the author of the English-language version or a non-English translation of a risk assessment tool was associated with higher rates of predictive validity. The review concluded that studies on which the author or translator of an actuarial tool was also a study author produced similar AUCs to studies conducted by independent investigations. These findings were replicated for SCJ tools. However, the justification for these conclusions lied in overlapping 95% confidence intervals, which are not equivalent to formal significance tests [17]. As with the previous review, another problem with this review is the use of the AUC, which has been criticized for offering overly-optimistic interpretations of the abilities of risk assessment tools to accurately predict violent behavior [18], [19]. Furthermore, the AUC can also not be used to conduct meta-regression, an extension of subgroup analysis which allows the effect of continuous as well as categorical characteristics to be investigated at a given significance level [20]. Thus, it may be that Guy's findings are false negatives.

### The Present Review

Given the limitations of previous reviews and their contrasting findings, the aim of the present systematic review and meta-analysis was to explore the evidence for an authorship effect using subgroup analyses and metaregression in a broader range of commonly used risk assessment tools, looking at published and unpublished literature. The independence of any authorship bias from other design-related moderators will also be explored, as will the role of translators of instruments.

## Methods

### Review Protocol

The Preferred Reporting Items for Systematic Reviews and Meta-analyses (PRISMA) Statement [21], a 27-item checklist of review characteristics designed to enable a transparent and consistent reporting of results (Table S1), was followed.

### Risk Assessment Tools

The following nine instruments were identified as those most commonly used in clinical practice based on recent questionnaire surveys [22]–[24] and reviews: the *Historical, Clinical, Risk Management-20* (HCR-20) [25], [26], the *Level of Service Inventory-Revised* (LSI-R) [27], the *Psychopathy Checklist-Revised* (PCL-R) [28], [29], the *Spousal Assault Risk Assessment* (SARA) [30]–[32], the *Structured Assessment of Violence Risk in Youth* (SAVRY) [33]–[34], the *Sex Offender Risk Appraisal Guide* (SORAG) [10], [11], the *Static-99* [12], [13], the *Sexual Violence Risk-20* (SVR-20) [35], and the *Violence Risk Appraisal Guide* (VRAG) [10], [11]. Details of these instruments are reported in Table 1.

**Table 1:** Characteristics of nine commonly used violence risk assessment tools.

Instrument	Approach	Outcome
LSI-R	Actuarial	General Offending
PCL-R	Actuarial	N/A^a^
SORAG	Actuarial	Violent Offending
Static-99	Actuarial	Violent + Sexual Offending
VRAG	Actuarial	Violent Offending
HCR-20	SCJ	Violent Offending
SARA	SCJ	Violent Offending
SAVRY	SCJ	Violent Offending
SVR-20	SCJ	Violent + Sexual Offending

*Note*. SCJ  =  structured clinical judgment; N/A  =  not applicable.

a The PCL-R was designed as a personality measure rather than a risk assessment tool, but is frequently used as means to assess risk of violent, sexual and general offending.

### Systematic Search

A systematic search was conducted to identify predictive validity studies for the above nine risk assessment tools using PsycINFO, EMBASE, MEDLINE, and US National Criminal Justice Reference Service Abstracts and the acronyms and full names of the instruments as keywords. Additional studies were identified through references, annotated bibliographies, and correspondence with researchers in the field known to us to be experts. Both peer-reviewed journal articles and unpublished investigations (i.e., doctoral dissertations, Master's theses, government reports, and conference presentations) from all countries were considered for inclusion. Manuscripts in all languages were considered, and there were no problems obtaining translations for non-English manuscripts. Studies measuring the predictive validity of select scales of an instrument were excluded, as were calibration studies because they may likely have produced inflated predictive validity estimates. When multiple studies used overlapping samples, that with the largest sample size was included to avoid double-counting.

Rates of true positives, false positives, true negatives, and false negatives at a given threshold (i.e., information needed to construct a two-by-two contingency table) needed to have been reported for a study to be included in the meta-analysis. When cut-off thresholds different to those suggested in the most recent version of a tool's manual were used to categorize individuals as being at low, moderate, or high risk of future offending, tabular data was requested from study authors. If the predictive validity of multiple instruments was assessed in the same study, data was requested for each tool and counted separately. Thus, one study could contribute multiple samples. In cases where different outcomes were reported, that with the highest base rate (i.e., the most sensitive) was selected.

**Figure 1:**
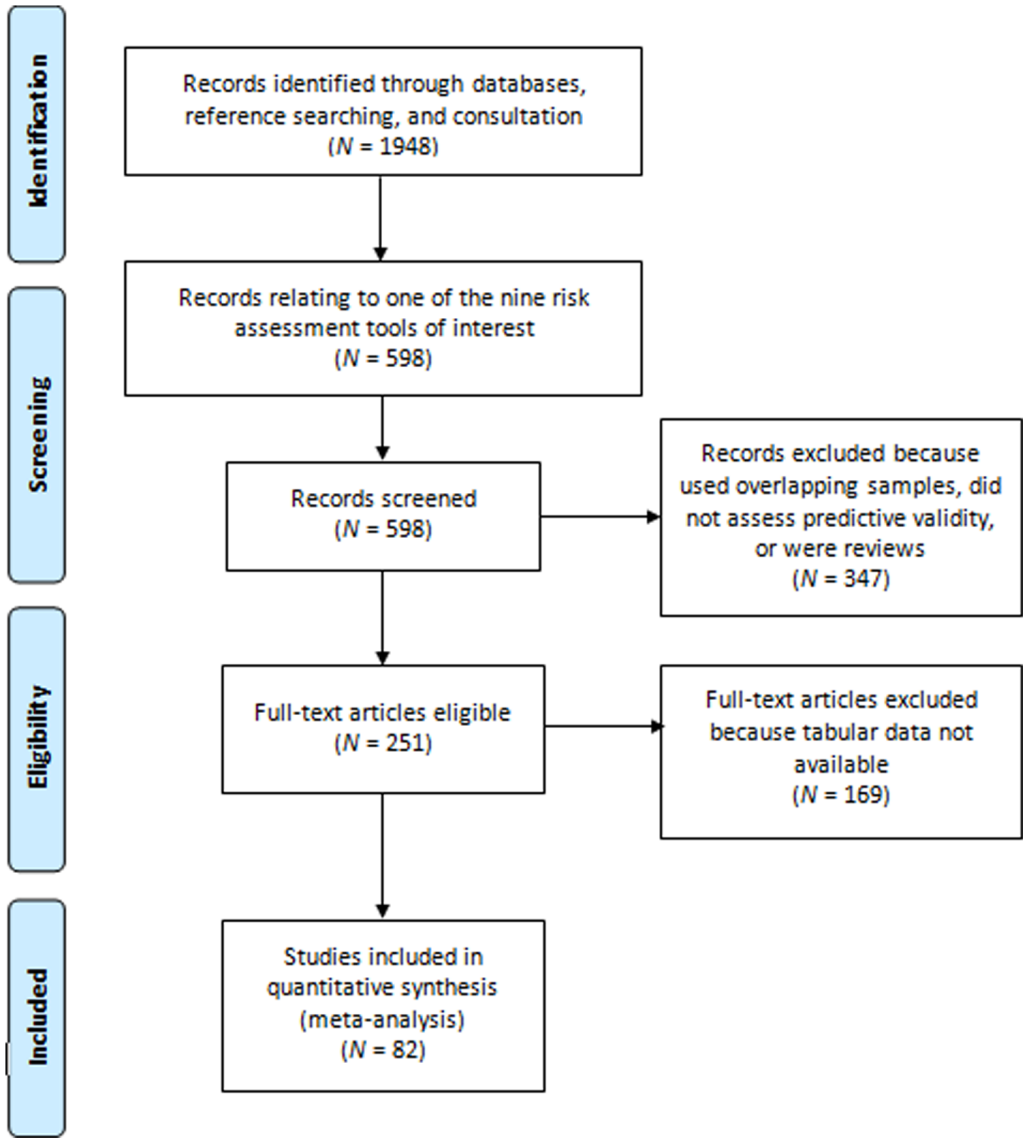
Results of a Systematic Search Conducted to Identify Replication Studies of Commonly Used Risk Assessment Tools.

Using this search strategy, 251 eligible studies were identified (Figure 1). Tabular data using standardized cut-off thresholds was available in the manuscripts of 31 of these studies (*k* samples = 39) and are thus available in the public domain. Additional data were requested from the authors of 164 studies (*k* = 320) and obtained for 52 studies (*k* = 65). The tabular data provided by the authors was based on further analysis of original datasets rather than analyses that had already been conducted, and was received after explaining to authors that the aim of the review to explore the predictive validity of commonly used risk assessment tools. Effect sizes from 234 of the 255 samples for which we were unable to obtain data were converted to Cohen's *d* using formulae published by Cohen [36], Rosenthal [37], and Ruscio [38]. The median effect size produced by those samples for which we could not obtain data (*Median* = 0.67; *Interquartile range* [*IQR*] = 0.45–0.87) and those for which we were able to obtain tabular data (*Median* = 0.74; *IQR* = 0.54–0.95) was similar, suggesting generalizability of the included samples.

### Data Analysis

As risk assessment instruments are predominantly used in clinical situations as tools for identifying higher risk individuals [39], participants who were classified as being at moderate or high risk for future offending were combined and compared with those classified as low risk for the primary analyses. A sensitivity analysis was conducted with participants classified as high risk compared to those classified as low or moderate risk. This second approach is more consistent with risk instruments being used for screening.

Six of the included instruments categorize individuals into one of three risk categories: low, moderate, or high risk. For the LSI-R, the low and low-moderate risk classifications were combined for the low risk category, and the moderate-high and high classifications were combined for the high risk category, leaving the moderate group unaltered. For the PCL-R, psychopathic individuals (scores of 30 and above) were considered the high risk group and non-psychopathic individuals were considered the low risk group, leaving no moderate risk bin. For the Static-99, the moderate-low and moderate-high classifications were combined and considered the moderate risk category, leaving the low and high groups unchanged.

Sufficient tabular data was available for the sensitivity analysis but not the primary analyses for 8 studies (*k* = 10). Therefore, data on 74 studies (*k* samples = 94) were included in the primary analyses, and data on 82 studies (*k* = 104) were included in the sensitivity analysis (references for included studies in List S1).

### Predictive Validity Estimation

The performance estimate used to measure predictive validity was the diagnostic odds ratio (DOR). The DOR is resistant to changes in the base rate of offending and may be easier to understand for non-specialists than alternative statistics such as the AUC or Pearson correlation coefficient, as it can be interpreted as an odds ratio. That is, the DOR is the ratio of the odds of a positive test result in an offender (i.e., the odds of a true positive) relative to the odds of a positive result in a non-offender (i.e., the odds of a false positive) at a given threshold [40]. The use of the DOR is currently considered as a standard approach when using metaregression methodology [40].

The Moses-Littenberg-Shapiro regression test [41] was used to determine whether DORs could be pooled. This standard test plots a measure of threshold against the natural log of each sample's odds ratio. As non-significant relationships were found between threshold and performance when those judged to be at moderate risk were considered high risk (*β* = –0.01, *p* = 0.32) or low risk (*β* = 0.01, *p* = 0.93), DerSimonian-Laird random effects meta-analysis was able to be performed using the sample DOR data. Between-study heterogeneity was measured using the *I^2^* index, which calculates the percentage of variation across samples not due to chance, and the *Q* statistic, which assesses the significance of variation across samples.

### Investigating the Presence of an Authorship Effect

Random effects subgroup analysis and meta-regression were used to explore evidence of an authorship effect. Tool designer status was operationally defined as being one of the authors of the English-language version of an included instrument. Further analyses were conducted to investigate the evidence for the authorship effect in studies of actuarial versus SCJ instruments, in studies published in a peer-reviewed journal versus gray literature (doctoral dissertations, Master's theses, government reports, and conference presentations), and when the definition of tool authorship was broadened to include translators of the instrument.

To investigate whether having a tool designer as a study author influenced predictive validity independently of other sample- and study-level characteristics, multivariable meta-regression was used to calculate unstandardized regression coefficients to test models composed of tool authorship and the type of offending being predicted (general vs. violent), ethnic composition (the percentage of a sample that was white), and the mean age of the sample (in years). These factors were previously been found to significantly moderate predictive validity estimates in published univariate analyses using a subset of the included studies [42]. The following indicators of methodological quality were then investigated in bivariate model with tool authorship to investigate moderating effects: temporal design (prospective versus not), inter-reliability of risk assessment tool administration, the training of tool raters (trained in use of the tool under investigation versus not), the professional status of tool raters (students versus clinicians), and whether outcomes were cross-validated (e.g., conviction versus self-report).

A standard significance level of α = 0.05 was adopted for these analyses, which were conducted using STATA/IC Version 10.1 for Windows. We have tested the accuracy of these tools in predicting violence, sexual violence, and criminal offending more generally in a related publication [43].

## Results

### Descriptive Characteristics

The present review included 30,165 participants in 104 samples from 83 independent studies. Information from 65 (*n* = 18,343; 62.5%) of the samples was not available in manuscripts and was received from study authors for the purposes of this synthesis. Of the 30,165 participants in the included samples, 9,328 (30.9%) offended over an average of 53.7 (*SD* = 40.7) months (Table 2). The tools with the most samples included the PCL-R (*k* = 21; 21.2%), the Static-99 (*k* = 18; 17.3%), and the VRAG (*k* = 14; 13.5%). The majority of the samples (*k* = 72; 69.2%) were assessed using an actuarial instrument. As suggested by Cicchetti [44], acceptable inter-rater reliability estimates were reported in all 56 (53.8%) samples on which agreement was investigated. Training in the risk assessment tool under investigation was reported for 37 (35.6%) of samples. Graduate students administered risk assessment tools in 19 (18.3%) samples, clinicians in 33 (31.7%), and a mix of both students and clinicians in 8 (7.7%). It was unstated or unclear for the remaining 48 (46.2%) samples who conducted assessments. Outcomes were cross-validated for three (2.9%) samples. Given the lack of information on the educational level of tool raters as well as the low prevalence of outcome cross-validation, these variables were excluded from subsequent metaregression analyses.

**Table 2:** Characteristics of 104 replication samples investigating the predictive validity of risk assessment tools.

		All samples	Designers' research	Independent research
Category	Subcategory	Number of *k* = 104 (%)	Number of *k* = 12 (%)	Number of *k* = 92 (%)
Source of study	Journal article	80 (76.9)	10 (83.3)	70 (76.1)
	Conference	7 (6.7)	2 (16.7)	5 (5.4)
	Thesis or dissertation	13 (12.5)	0 (0)	13 (14.1)
	Government report	4 (3.8)	0 (0)	4 (4.3)
Type of tool	Actuarial	72 (69.2)	6 (50.0)	66 (71.7)
	SCJ	32 (30.8)	6 (50.0)	26 (28.3)
Tool used	HCR-20	12 (11.4)	2 (16.7)	10 (10.9)
	LSI-R	11 (10.6)	0 (0)	11 (12.0)
	PCL-R	21 (21.2)	0 (0)	21 (22.8)
	SARA	4 (3.8)	3 (25.0)	1 (1.1)
	SAVRY	11 (10.6)	1 (8.3)	10 (10.9)
	SORAG	8 (7.7)	2 (16.7)	6 (6.5)
	Static-99	18 (17.3)	2 (16.7)	16 (17.4)
	SVR-20	5 (4.8)	0 (0)	5 (5.4)
	VRAG	14 (13.5)	2 (16.7)	12 (13.0)
Sample size	Mean (*SD*)	366 (513)	449 (725)	356 (483)
Male participants (per sample)	Mean % (*SD*)	95.3 (14.6)	99.3 (2.6)	94.6 (15.7)
White participants (per sample)	Mean % (*SD*)	72.7 (22.8)	75.1 (9.9)	72.4 (24.1)
Age (in years)	Mean (*SD*)	32.1 (7.6)	33.4 (8.4)	40.2 (2.2)
Study setting^a^	Community	4 (3.8)	2 (16.7)	2 (2.2)
	Correctional	44 (42.3)	5 (41.7)	39 (42.4)
	Psychiatric	41 (39.4)	3 (25.0)	38 (41.3)
	Mixed	15 (14.4)	2 (16.7)	13 (14.1)
Temporal design	Prospective	46 (44.2)	5 (41.7)	41 (44.6)
	Retrospective	53 (51.0)	5 (41.7)	48 (52.2)
	Unstated/Unclear	5 (4.8)	2 (16.7)	3 (3.3)
Source of information used to	File review	60 (57.7)	8 (66.7)	52 (56.5)
administer tool	Interview	2 (1.9)	0 (0)	2 (2.2)
	Mixed	20 (19.2)	3 (25.0)	17 (18.5)
	Unstated/Unclear	22 (21.2)	1 (8.3)	21 (22.8)
Length of follow-up (months)	Mean (*SD*)	53.7 (40.7)	50.3 (21.4)	97.4 (35.1)
Type of offending	General^b^	54 (51.9)	3 (25.0)	51 (55.4)
	Violent only	48 (46.2)	9 (75.0)	39 (42.4)
	Non-violent only	1 (1.0)	0 (0)	1 (1.1)
	Unstated/Unclear	1 (1.0)	0 (0)	1 (1.1)
Type of outcome	Charge/Arrest/Conviction	69 (66.4)	8 (66.7)	61 (66.3)
	Institutional incident	12 (11.5)	1 (8.3)	11 (12.0)
	Mixed	17 (15.3)	3 (25.0)	14 (15.2)
	Unstated/Unclear	6 (5.8)	0 (0)	6 (6.5)

*Note*. k =  number of samples; SCJ  =  structured clinical judgment; SD  =  standard deviation. Designer status operationally defined as being an author of the English-language original version of the instrument under investigation.

a At start of follow-up; ^b^Violent and non-violent.

A designer or translator of a risk assessment tool was also an author on a research study on that instrument in 25 (*k* = 29; 27.9%) of the 83 studies. Authors of the English-language version of a given tool's manual were also authors of a study investigating that tool's predictive validity on 10 studies constituting 12 (11.5%) samples: 3 (2.9%) samples for the SARA, 2 (1.9%) for the HCR-20, 2 (1.9%) for the SORAG, 2 (1.9%) for the Static-99, 2 (1.9%) for the VRAG, and 1 (1.0%) for the SAVRY. A tool's translator was also an author of a study investigating that tool's predictive validity in 15 studies constituting 17 (16.3%) samples: 4 (3.8%) samples for the Static-99, 3 (2.9%) for the HCR-20, 3 (2.9%) for the SVR-20, 2 (1.9%) for the PCL-R, 2 (1.9%) for the SAVRY, 2 (1.9%) for the SORAG, and 1 (1.0%) for the VRAG.

Six of the 16 journals in which the studies appeared requested in their Instructions for Authors that any financial or non-financial conflicts of interest be disclosed. None of the 25 studies where a tool designer or translator was the author of an investigation of that instrument's predictive validity contained such a disclosure.

### Investigation of an Authorship Effect

Random effects subgroup analysis found an authorship effect: higher predictive validity estimates were produced where study authors were also designers of the tool being investigated (DOR = 6.22; 95% CI = 4.68–8.26; *I^2^* = 0.0; *Q* = 3.94, *p* = 0.95) compared to independent studies (DOR = 3.08; 95% CI = 2.45–3.88; *I^2^* = 82.3; *Q* = 462.81, *p*<0.001) (Table 3). Metaregression confirmed this significant finding (*β = *0.83, *p* = 0.02). Although there was no clear evidence of the authorship effect in actuarial and SCJ instruments when considered separately (*β*_Actuarial_ = 0.78, *SE* = 0.48, *p* = 0.11; *β*_SCJ_ = 0.59, *SE* = 0.51, *p* = 0.26), there was evidence that studies of SCJ instruments conducted by teams not including a tool author or translator produced significantly higher DORs than studies of actuarial instruments (*β* = 0.68, *SE* = 0.27, *p* = 0.02). The authorship effect was specific to studies published in a peer-reviewed journal (*β* = 0.79, *SE* = 0.38, *p* = 0.04) rather than doctoral dissertations, Master's theses, government reports, and conference presentations (*β* = –1.03, *SE* = 1.05 *p* = 0.34). When the operational definition of tool authorship was broadened to include translators, a non-significant trend towards an authorship effect was found (*β* = 0.39, *SE* = 0.26, *p* = 0.13).

**Table 3:** Subgroup and metaregression analyses of diagnostic odds ratios (DORs) produced by nine commonly used risk assessment tools when a tool designer was a study author versus independent investigations.

Analysis	Subcategory	Authorship status	*DOR* (95% CI)	Metaregression
Overall	Translators not	Tool designer as study author	6.22 (4.68–8.26)	*β* = 0.83, *SE* = 0.36, *p* = 0.02
	included as “designers”	Tool designer not study author	3.08 (2.45–3.88)	
	Translators included	Tool designer as study author	4.45 (3.06–6.47)	*β* = 0.39, *SE* = 0.26, *p* = 0.13
	as “designers”	Tool designer not study author	3.04 (2.36–3.91)	
Type of	Actuarial	Tool designer as study author	5.38 (3.82–7.58)	*β* = 0.78, *SE* = 0.48, *p* = 0.11
tool^a^		Tool designer not study author	2.56 (1.98–3.30)	
	SCJ	Tool designer as study author	8.60 (5.15–14.35)	*β* = 0.59, *SE* = 0.51, *p* = 0.26
		Tool designer not study author	5.07 (3.27–7.84)	
Publication	Journal	Tool designer as study author	6.13 (4.59–8.20)	*β* = 0.79, *SE* = 0.38, *p* = 0.04
source^a^		Tool designer not study author	3.09 (2.39–3.98)	
	Gray literature	Tool designer as study author	8.73 (2.06–36.94)	*β* = –1.03, *SE* = 1.05, *p* = 0.34
		Tool designer not study author	3.07 (1.93–4.90)	

*Note. β* =  unstandardized regression coefficient; SE  =  standard error; SCJ  =  structured clinical judgment; DOR  =  diagnostic odds ratio; CI  =  confidence interval; Gray literature  =  doctoral dissertations, Master's theses, government reports, and conference presentations.

a Authorship operationally defined as being an author of the English-language version of the instrument under investigation.

Multivariable meta-regression was used to investigate whether having a tool designer as a study author influenced predictive validity independently of other sample- and study-level characteristics including the type of offending being predicted (*β* = –0.01, *SE* = 0.52, *p* = 0.99), ethnic composition (*β* = 0.02, *SE* = 0.01, *p* = 0.08), and the mean age of the sample (*β* = –0.03, *SE* = 0.02, *p* = 0.22). When these variables were modeled together, tool authorship remained a borderline significant predictor of predictive validity (*β* = 1.02, *SE* = 0.55, *p* = 0.08). Bivariate models revealed that methodological quality indicators including temporal design (*β* = 0.11, *SE* = 0.27, *p* = 0.68), inter-rater reliability (*β* = 0.02, *SE* = 0.24, *p* = 0.94), training of tool raters (*β* = 0.04, *SE* = 0.23, *p* = 0.87), and professional status (*β* = 0.23, *SE* = 0.34, *p* = 0.51) did not account for variance in predictive validity estimates independently of tool authorship, which remained significant at the *p*<0.05 level throughout. Whether outcomes were cross-validated was not able to be investigated due to low cell counts.

### Sensitivity Analysis

No clear evidence of an authorship effect was found when moderate risk individuals were grouped with low risk participants and authorship was operationally defined as being an author of the English-language version of an instrument (*β* = 0.35, *p* = 0.31) or an author of either an English-language or translated version (*β* = –0.10, *p* = 0.67).

## Discussion

Violence risk assessment is increasingly part of routine clinical practice in mental health and criminal justice systems. The present meta-analysis examined if an authorship effect exists in the violence risk assessment literature, namely whether studies in which a designer of one of these tools was also a study author found more favorable predictive validity results than independent investigations. To explore this, tabular data was obtained for 30,165 participants in 104 samples from 83 independent studies. We report two main findings: evidence of an authorship effect, and clear lack of disclosure. Both have potentially important implications for the field.

Evidence of a significant authorship effect was found, specifically to risk assessment studies published in peer-reviewed journals. Previous work has proposed several possible explanations of such bias [7], [9], [45]. First, tool designers may conduct studies to maximize the predictive validity of their instruments. Such biases may be incidental as tool designers are more familiar with their instrument, might be more careful to ensure proper training of tool administrators, and promote use following manualized protocols without modification. The involvement of tool designers may result in experimenter effects that influence assessors. Such effects may encourage clinicians to adhere more closely to protocols, which is likely associated with better performance and fidelity [42]. That the authorship effect appeared to be more pronounced in studies of actuarial instruments may be attributed to this–actuarial instruments have stricter administration protocols which, if not followed exactly, may result in considerably different predictive validity estimates [46]. This finding will need replication with larger datasets to clarify, however.

A second potential reason for the authorship effect is that tool designers may be unwilling to publish studies where their instrument performs poorly. Such a “file drawer problem” [47] is well established in other fields, especially where a vested interest is involved [48] and supports the recent call for prospective registration of observational research [49]. Given that multivariable analyses suggested that the authorship effect might be confounded by the type of offense being predicted and samples' ethnic composition and mean age, a third reason for the authorship effect may be that tool authorship represents a proxy for having used a risk assessment tool as it was designed to be used (e.g. to predict violent offending in psychiatric patients) in samples similar to that tool's development sample (e.g. in youths, or predominantly white individuals in their late 20s and early 30s). However, we found no evidence that the authorship effect was related to methodological quality indicators such as inter-rater reliability or training in the use of the instrument under investigation. Whatever the possible reasons, this is an important finding for the field, with implications for research, clinical practice, and the interaction of forensic mental health with the criminal justice field. For example, the suitability of candidates for expert panels and task forces for reviewing evidence, writing clinical guidelines, and setting up policy documents, needs to consider authorship effects. Similarly, potential conflicts of interests in expert witness work in legal cases need declaration.

Limitations of the present review include the fact that we did not have access to information from all relevant studies, and that we focused our review on what are the most commonly used instruments and therefore did not included some newer instruments. We used as the outcome with the highest base rate for a particular instrument, because analyses of the authorship effect by class of tools (those designed for violence, sexual violence, or criminal offending) were underpowered. We were also unable to conduct analyses by individual instruments, as there were three or fewer studies with tool authors as study authors for each. Finally, we did not have access to sufficient details on each study to systematically assess further if the authorship effect was linked to fidelity in designers' research, such as information about raters' training, inter-rater reliability of tool items, or cross-validation of outcome measures.

As there was evidence of an authorship bias, the financial and non-financial benefits of authors warrant disclosure in this field, particularly when a journal's *Instructions to Authors* request that any potential conflicts of interest be made clear. Such disclosure has been established as a first step towards dealing with conflicts of interest in psychiatry [50]. The present meta-analysis found that such transparency has yet to have been achieved in the forensic risk assessment literature. None of the 25 studies where tool authors or translators were also study authors reported a conflict of interest, despite 6 of the 16 journals in which they were published having requested that potential conflicts be disclosed. The number of journals requesting such disclosures may higher, as information requested not in in Instructions to Authors but rather during the manuscript submission process was not investigated. Apparent lack of compliance with guidelines may have due to journals choosing not to publish a disclosure made by study authors or study authors may have decided not to report their financial and/or non-financial interests [51]. To promote transparency in future research, tool authors and translators should routinely report their potential conflict of interest when publishing research investigating the predictive validity of their tool.

## Conclusions

Conflicts of interest are an important area of investigation in medical and behavioral research, particularly as there has been concern about trial data being influenced by industry sponsorship. Having explored this issue in the growing violence risk assessment literature, we have found evidence of both an authorship effect as well as lack of disclosure by tool designers and translators. The credibility of future research findings may be questioned in the absence of measures to tackle these issues [50], [52]. Further, when assessing the suitability of candidates for expert panels and task forces for reviewing evidence, writing clinical guidelines, and setting up policy documents, it is pertinent to consider authorship effects. Similarly, potential conflicts of interests in expert witness work in legal cases need declaration.

## Supporting Information

Table S1 **Preferred Reporting Items for Systematic Reviews and Meta-analyses (PRISMA) Statement.**
(DOC)

List S1 **References of studies included in meta-analysis.**
(DOC)

## References

[1] Lexchin J, Bero LA, Djulbegovic B, Clark O (2003) Pharmaceutical industry sponsorship and research outcome and quality: Systematic review. BMJ 326: 1167–1170. 12775614 10.1136/bmj.326.7400.1167 PMC156458

[2] Perlis RH, Perlis CS, Wu Y, Hwang C, Joseph M, et al (2005) Industry sponsorship and financial conflict of interest in the reporting of clinical trials in psychiatry. Am J Psychiatry 162: 1957–1960. 16199844 10.1176/appi.ajp.162.10.1957

[3] Bekelman JE, Li Y, Gross CP (2003) Scope and impact of financial conflicts of interest in biomedical research: A systematic review. JAMA 289: 454–465. 12533125 10.1001/jama.289.4.454

[4] Singh JP (2012) The history, development, and testing of forensic risk assessment tools. In: Grigorenko E, editor. Handbook of juvenile forensic psychology and psychiatry. New York: Springer.

[5] Singh JP, Fazel S (2010) Forensic risk assessment: A metareview. Crim Justice Behav 37: 965–988.

[6] Monahan J (1981) The clinical prediction of violent behavior. Washington, DC: Government Printing House.

[7] Blair PR, Marcus DK, Boccaccini MT (2008) Is there an allegiance effect for assessment instruments? Actuarial risk assessment as an exemplar. Clinical Psychol 15: 346–360.

[8] Guy L (2008) Performance indicators of the structured professional judgement approach for assessing risk for violence to others: A meta-analytic survey. Burnaby, BC: Simon Fraser University.

[9] Harris GT, Rice ME, Quinsey VL (2010) Allegiance or fidelity? A clarifying reply. Clinical Psychol 17: 82–89.

[10] Quinsey VL, Harris GT, Rice ME, Cormier CA (1998) Violent offenders: Appraising and managing risk. Washington, DC: American Psychological Association.

[11] Quinsey VL, Harris GT, Rice ME, Cormier CA (2006). Violent offenders: Appraising and managing risk (2nd ed.). Washington, DC: American Psychological Association.

[12] Hanson RK, Thornton D (1999) Static-99: Improving actuarial risk assessments for sex offenders. Ottawa, ON: Department of the Solicitor General of Canada.

[13] Harris AJR, Phenix A, Hanson RK, Thornton D (2003) Static-99 coding rules: Revised 2003. Ottawa, ON: Solicitor General Canada.

[14] Marzban C (2004) The ROC curve and the area under it as performance measures. Weather & Forecasting 19: 1106–1114.

[15] Ware JH (2006) The limitations of risk factors as prognostic tools. NEJM 355: 2615–2617. 17182986 10.1056/NEJMp068249

[16] Vickers AJ, Cronin AM, Begg CB (2011) One statistical test is sufficient for assessing new predictive markers. BMC Med Res Methodol 11: 1–7. 21276237 10.1186/1471-2288-11-13 PMC3042425

[17] Belia S, Fidler F, Williams J, Cumming G (2005) Researchers misunderstand confidence intervals and standard error bars. Psychol Meth 10: 389–396. 10.1037/1082-989X.10.4.389 16392994

[18] Singh JP (2013) Predictive validity performance indicators in violence risk assessment: A methodological primer. Behav Sci Law, doi:10.1002/bsl.2052. 10.1002/bsl.2052 23408459

[19] Sjöstedt G, Grann M (2002) Risk assessment: What is being predicted by actuarial prediction instruments? Int J Forensic Ment Health 1: 179–183.

[20] Morton SC, Adams JL, Suttorp MJ, Shekelle PG (2004) Meta-regression approaches: What, why, when, and how? Rockville, MD: Agency for Healthcare Research and Quality.

[21] Moher D, Liberati A, Tetzlaff J, Altman DG (2009) Preferred reporting items for systematic reviews and meta-analyses: The PRISMA Statement. PLoS Med 6: e1000097. 19621072 10.1371/journal.pmed.1000097 PMC2707599

[22] Archer RP, Buffington-Vollum JK, Stredny RV, Handel RW (2006) A survey of psychological test use patterns among forensic psychologists. J Pers Assess 87: 84–94. 16856789 10.1207/s15327752jpa8701_07

[23] Khiroya R, Weaver T, Maden T (2009) Use and perceived utility of structured violence risk assessments in English medium secure forensic units. Psychiatrist 33: 129–132.

[24] Viljoen JL, McLachlan K, Vincent GM (2010) Assessing violence risk and psychopathy in juvenile and adult offenders: A survey of clinical practices. Assessment 17: 377–395. 20124429 10.1177/1073191109359587

[25] Webster CD, Douglas KS, Eaves D, Hart SD (1997) HCR-20: Assessing risk for violence. Version 2. Burnaby, BC: Simon Fraser University, Mental Health, Law, and Policy Institute.

[26] Webster CD, Eaves D, Douglas KS, Wintrup A (1995) The HCR-20 scheme: The assessment of dangerousness and risk. Vancouver, BC: Mental Health Law and Policy Institute, and Forensic Psychiatric Services Commission of British Columbia.

[27] Andrews DA, Bonta J (1995) LSI-R: The Level of Service Inventory-Revised. Toronto, ON: Multi-Health Systems.

[28] Hare RD (1991) The Hare Psychopathy Checklist-Revised. North Tonawanda, NY: Multi-Health Systems.

[29] Hare RD (2003) The Hare Psychopathy Checklist-Revised (2nd ed.). Toronto, ON: Multi-Health Systems.

[30] Kropp PR, Hart SD, Webster CD, Eaves D (1994) Manual for the Spousal Assault Risk Assessment guide. Vancouver, BC: British Columbia Institute on Family Violence.

[31] Kropp PR, Hart SD, Webster CD, Eaves D (1995) *Manual for the Spousal Assault Risk Assessment guide* (2nd ed.). Vancouver, BC: British Columbia Institute on Family Violence.

[32] Kropp PR, Hart SD, Webster CD, Eaves D (1999) Spousal Assault Risk Assessment guide (SARA). Toronto, ON: Multi-Health Systems.

[33] Borum R, Bartel P, Forth A (2003) Manual for the Structured Assessment of Violence Risk in Youth (SAVRY). Version 1.1. Tampa, FL: University of South Florida.

[34] Borum R, Bartel P, Forth A (2002) Manual for the Structured Assessment of Violence Risk in Youth (SAVRY). Tampa, FL: University of South Florida.

[35] Boer DP, Hart SD, Kropp PR, Webster CD (1997) Manual for the Sexual Violence Risk-20. Professional guidelines for assessing risk of sexual violence. Burnaby, BC: Simon Fraser University, Mental Health, Law, and Policy Institute.

[36] Cohen J (1988) Statistical power analysis for the behavioral sciences (2nd ed.). Hillsdale, NJ: Erlbaum.

[37] Rosenthal R (1994) Parametric measures of effect size. In: Cooper H, Hedges LV, editors. The handbook of research synthesis. New York: Sage.

[38] Ruscio J (2008) A probability-based measure of effect size: Robustness to base rates and other factors. Psychol Meth 13: 19–30. 10.1037/1082-989X.13.1.19 18331151

[39] Buchanan A, Leese M (2001) Detention of people with dangerous severe personality disorders: A systematic review. Lancet 358: 1955–1959. 11747920 10.1016/S0140-6736(01)06962-8

[40] Egger M, Smith GD, Altman D (2001) Systematic reviews in health care: Meta-analysis in context. London: BMJ Publishing Groups.

[41] Moses LE, Littenberg B, Shapiro D (1993) Combining independent studies of a diagnostic test into a summary ROC curve: Data-analytical approaches and some additional considerations. Stat Med 12: 1293–1316. 8210827 10.1002/sim.4780121403

[42] Singh JP, Grann M, Fazel S (2011) A comparative study of risk assessment tools: A systematic review and metaregression analysis of 68 studies involving 25,980 participants. Clin Psychol Rev 31: 499–513. 21255891 10.1016/j.cpr.2010.11.009

[43] Fazel S, Singh JP, Doll H, Grann M (2012) Use of risk assessment instruments to predict violence and antisocial behaviour in 73 samples involving 24,827 people: Systematic review and meta-analysis. BMJ 345: e4692. 22833604 10.1136/bmj.e4692 PMC3404183

[44] Cicchetti DV (2001) The precision of reliability and validity estimates re-visited: Distinguishing between clinical and statistical significance of sample size requirements. J Clin Exp Neuropsychol 23: 695–700. 11778646 10.1076/jcen.23.5.695.1249

[45] Lilienfeld SO, Jones MK (2008) Allegiance effects in assessment: Unresolved questions, potential explanations, and constructive remedies. Clinical Psychol 15: 361–365.

[46] Harris GT, Rice ME (2003) Actuarial assessment of risk among sex offenders. Ann NY Acad Sci 989: 198–210. 12839899 10.1111/j.1749-6632.2003.tb07306.x

[47] Rosenthal R (1979) The “file drawer problem” and the tolerance for null results. Psychol Bull 86: 638–641.

[48] Thompson D (1993) Understanding financial conflicts of interest. NEJM 329: 573–576. 8336759 10.1056/NEJM199308193290812

[49] Editorial (2010) Should protocols for observational research be registered? Lancet 375: 348. 10.1016/S0140-6736(10)60148-1 20113809

[50] Fava GA (2009) An operational proposal for addressing conflict of interest in the psychiatric field. J Ethics Ment Health 4: S1–S5.

[51] Krimsky S, Rothenberg LS (2001) Conflict of interest policies in science and medical journals: Editorial practices and author disclosures. Sci Eng Ethics 7: 205–218. 11349360 10.1007/s11948-001-0041-7

[52] Maj M (2008) Non-financial conflicts of interests in psychiatric research and practice. Br J Psychiatry 193: 91–92. 18669986 10.1192/bjp.bp.108.049361

