# Supplementary material for: Authorship Bias in Violence Risk Assessment? A Systematic Review and Meta-Analysis
Source: PLoS One. 2013 Sep 2;8(9):e72484. doi: 10.1371/journal.pone.0072484 (PMC3759386; doi:10.1371/journal.pone.0072484)
Supplement: List S1 — References of studies included in meta-analysis. (DOC) [file pone.0072484.s002.doc]

**List S1.** References of studies included in meta-analysis

1. Arbach K, Pueyo AA (2007) Violence risk assessment in mental disorders with the HCR-20. Papeles del Psicologo 28: 174-186.
2. Austin J, Coleman D, Peyton J, Johnson KD (2003) Reliability and validity study of the LSI-R risk assessment instrument. Washington, DC: Pennsylvania Board of Probation and Parole.
3. Beggs SM, Grace RC (2008) Psychopathy, intelligence, and recidivism in child molesters: Evidence of an interaction effect. Crim Justice Behav 35: 683-695.
4. Bengtson S (2008) Is newer better? A cross-validation of the Static-2002 and the Risk Matrix 2000 in a Danish sample of sexual offenders. Psychol Crim Law 14: 85-106.
5. Caperton JD (2005) Predicting recidivism among sex offenders: Utility of the STATIC-99, Minnesota Sex Offender Screening Tool-Revised, and Psychopathy Checklist-Revised. Huntsville, TX: Sam Houston State University.
6. Dahle KP (2006) Strengths and limitations of actuarial prediction of criminal reoffense in a German prison sample: A comparative study of LSI-R, HCR-20 and PCL-R. Int J Law Psychiatr 29: 431-442.
7. Davidson J (2007) Risky business: What standard assessments mean for female offenders. Manoa, University of Hawaii.
8. de Vogel V, de Ruiter C (2005) The HCR-20 in personality disordered female offenders: A comparison with a matched sample of males. Clin Psychol Psychother 12: 226-240.
9. de Vogel V, de Ruiter C, van Beek, Mead G (2004) Predictive validity of the SVR-20 and Static-99 in a Dutch sample of treated sex offenders. Law Hum Behav 28: 235-251.
10. Dempster RJ (1998) Prediction of sexually violent recidivism: A comparison of risk assessment instruments. Burnaby, BC: Simon Fraser University.
11. Dempster RJ (2001) Understanding errors in risk assessment: The application of differential prediction methodology. Burnaby, BC: Simon Fraser University.
12. Dolan M, Blattner R (2010) The utility of the Historical Clinical Risk-20 Scale as a predictor of outcomes in decisions to transfer patients from high to lower levels of security: A UK perspective. BMC Psychiatry 10: 1-8.
13. Dolan M, Rennie CE (2008) The Structured Assessment of Violence Risk in Youth as a predictor of recidivism in a United Kingdom cohort of adolescent offenders with conduct disorder. Psychol Asses 20: 35-46.
14. Douglas KS, Ogloff JRP, Hart SD (2003) Evaluation of a model of violence risk assessment among forensic psychiatric patients. Psychiatr Serv 2003 54: 1372-1379.
15. Douglas KS, Yeomans M, Boer DP (2005) Comparative validity analysis of multiple measures of violence risk in a sample of criminal offenders. Crim Justice Behav 32: 479-510.
16. Dowdy ER, Lacy MG, Unnithan NP (2002) Correctional prediction and the Level of Supervision Inventory. J Crim Justice 30: 29-39.
17. Ducro C, Pham T (2006) Evaluation of the SORAG and the Static-99 on Belgian sex offenders committed to a forensic facility. Sex Abuse 18: 15-26.
18. Eher R, Rettenberger M, Matthes A (2009) Aktuarische prognose bei Sexualstraftaetern. [Actuarial predictions of sexual offenders]. Mschr Krim 92: 18-27.
19. Eher R, Rettenberger M, Schilling F, Pfäfflin F (2008) Failure of Static-99 and SORAG to predict reoffense categories in relevant sexual offender subtypes: a prospective study. Sex Offend Treat 3: 1-14.
20. Fass TL, Heilbrun K, DeMatteo D, Fretz R (2008) The LSI-R and the COMPAS: Validation data on two risk-needs tools. Crim Justice Behav 35: 1095-1108.
21. Folino J (2006) Estudio de cohorte psiquiatrico: Factores de riesgo de violencia. [Psychiatric cohort study: Risk factors for violence]. La Plata: La Sociedad Médica de la Plata y de Fundación.
22. Folino J, Almiron M, Ricci MA (2007) Factores de riesgo de recidiva violenta en mujeres filicidas. [Violent recidivism risk factor in filicidal women]. Vertex 18: 258-267.
23. Friendship C, Mann RE, Beech AR (2003) Evaluation of a national prison-based treatment program for sexual offenders in England and Wales. J Interpers Viol 18: 744-759.
24. Gammelgård M, Koivisto AM, Eronen M, Kaltiala-Heino R (2008) The predictive validity of the Structured Assessment of Violence Risk in Youth (SAVRY) among institutionalised adolescents. J Forensic Psychiatr Psychol 19: 352-370.
25. Gibas AL, Kropp PR, Hart SD, Stewart L (2008) Validity of the SARA in a Canadian sample of incarcerated adult males. Vienna, International Association of Forensic Mental Health Services, 2008.
26. Grann M, Belfrage H, Tengström A (2000) Actuarial assessment of risk for violence: Predictive validity of the VRAG and the historical part of the HCR-20. Crim Justice Behav 27: 97-114.
27. Grann M, Långström N, Tengström A, Kullgren G (1999) Psychopathy (PCL-R) predicts violent recidivism among criminal offenders with personality disorders in Sweden. Law Hum Behav 23: 205-217.
28. Gray NS, Snowden RJ, MacCulloch S, Phillips H, Taylor J, et al. (2004) Relative efficacy of criminological, clinical, and personality measures of future risk of offending in mentally disordered offenders: A comparative study of HCR-20, PCL:SV, and OGRS. J Consult Clin Psychol 72: 523-530.
29. Gretton H, Abramowitz C (2002) SAVRY: Contribution of items and scales to clinical risk judgments and criminal outcomes. Austin, TX: American Psychology-Law Society.
30. Harris GT, Rice ME, Quinsey VL, Lalumiere ML, Boer D, et al. (2003) A multisite comparison of actuarial risk instruments for sex offenders. Psychol Assess 15: 413-425.
31. Helmus LMD, Hanson RK (2007) Predictive validity of the Static-99 and Static-2002 for sex offenders on community supervision. Sex Offender Treat 2: 1-14.
32. Hill A, Habermann N, Klusmann D, Berner W, Briken P (2008) Criminal recidivism in sexual homicide perpetrators. Int J Offender Ther Comp Criminol 52: 5-20.
33. Ho H, Thomson L, Darjee R (2009) Violence risk assessment: The use of the PCL-SV, HCR-20, and VRAG to predict violence in mentally disordered offenders discharged from a medium secure unit in Scotland. J Forensic Psychiatr Psychol 4: 523-541.
34. Hollin CR, Palmer EJ (2006) The Level of Service Inventory-Revised profile of English prisoners: Risk and reconviction analysis. Crim Justice Behav 33: 347-366.
35. Jovanović AA, Toševski DL, Ivkonvić M, Damjanović A, Gašić MJ (2009) Predicting violence in veterans with posttraumatic stress disorder. Vojnosanit Pregl 66: 13-21.
36. Kelly CE, Welsh WN (2008) The predictive validity of the Level of Service Inventory-Revised for drug-involved offenders. Crim Justice Behav 35: 819-831.
37. Kingston DA, Yates PM, Firestone P, Babchishin K, Bradford JM (2008) Long-term predictive validity of the Risk Matrix 2000: A comparison with the Static-99 and the Sex Offender Risk Appraisal Guide. Sex Abuse 20: 466-484.
38. Kloezeman KC (2004) Violent behavior on inpatient psychiatric units: The HCR-20 violence risk assessment scheme. Manoa, HI: University of Hawaii.
39. Kroner C, Stadtland C, Eidt M, Nedopil N (2007) The validity of the Violence Risk Appraisal Guide (VRAG) in predicting criminal recidivism. Crim Behav Ment Health 17: 89-100.
40. Kropp PR, Hart SD (2000) The Spousal Assault Risk Assessment (SARA) guide: Reliability and validity in adult male offenders. Law Hum Behav 2000 24: 101-118.
41. Langton CM, Barbaree HE, Seto MC, Peacock EJ, Harkins L, et al. (2007) Actuarial assessment of risk for reoffense among adult sex offenders: Evaluating the predictive accuracy of the Static-2002 and five other instruments. Crim Justice Behav 34: 37-59.
42. Langton CM (2003) Contrasting approaches to risk assessment with adult male sexual offenders: An evaluation of recidivism prediction schemes and the utility of supplementary clinical information for enhancing predictive accuracy. Toronto, ON: University of Toronto.
43. Lodewijks HPB, Doreleijers TAH, de Ruiter C (2008) SAVRY risk assessment in violent Dutch adolescents: Relation to sentencing and recidivism. Crim Justice Behav 35: 696-709.
44. Lodewijks HPB, de Ruiter C, Doreleijers TAH (2008) Gender differences in violent outcome and risk assessment in adolescent offenders after residential treatment. Int J Forensic Ment Health 7: 105-141.
45. Lodewijks HPB, Doreleijers TAH, de Ruiter C, Borum R (2008) Predictive validity of the Structured Assessment of Violence Risk in Youth (SAVRY) during residential treatment. Int J Law Psychiatr 31: 263-271.
46. Looman J, Abracen J (2010) Comparison of measures of risk for recidivism in sexual offenders. J Interpers Viol 25: 791-807.
47. Manchak SM, Skeem JL, Douglas KS, Siranosian M (2009) Does gender moderate the predictive utility of the Level of Service Inventory Revised (LSI-R) for serious violent offenders. Crim Justice Behav 36: 425-442.
48. Manchak SM, Skeem JL, Douglas KS (2008) Utility of the revised Level of Service Inventory (LSI-R) in predicting recidivism after long-term incarceration. Law Hum Behav 32: 477-488.
49. McEachran A (2001) The predictive validity of the PCL:YV and the SAVRY in a population of adolescent offenders. Burnaby, BC: Simon Fraser University.
50. Meyers JR, Schmidt F (2008) Predictive validity of the Structured Assessment of Violence Risk in Youth (SAVRY) with juvenile offenders. Crim Justice Behav 35: 344-355.
51. Mills JF, Jones MN, Kroner DG (2005) An examination of the generalizability of the LSI-R and VRAG probability bins. Crim Justice Behav 32: 565-585.
52. Mills JF, Kroner DG (2006) The effect of discordance among violence and general recidivism risk estimates on predictive accuracy. Crim Behav Ment Health 16: 155-166.
53. Morrissey C, Hogue T, Mooney P, Allen C, Johnston S, et al. (2007) Predictive validity of the PCL-R in offenders with intellectual disability in a high secure hospital setting: Institutional aggression. J Forensic Psychiatr Psychol 18: 1-15.
54. Nicholls TL, Ogloff JRP, Ledwidge B (2007) Is the profound distrust of unbridled clinical opinion in the violence risk assessment field unfounded? Victoria, BC: International Association of Forensic Mental Health Services.
55. Pedersen L, Rasmussen K, Elsass P (2010) Risk assessment: The value of structured professional judgments. Int J Forensic Ment Health 9: 74-81.
56. Pham TH, Ducro C, Marghem B, Réveillère C (2005) Evaluation du risque de recidive au sein d'une population de delinquants incarceres ou internes en Belgique francophone. [Prediction of recidivism among prison inmates and forensic patients in Belgium]. Annales Medico Psychol 163: 842-845.
57. Pham TH, Ducro C, Marghem B (2005) Predicting recidivism among offenders populations in Belgium: The contribution of psychopathy to other risk assessment instruments. Louvain-la-Neuve: Universite Catholique de Louvain.
58. Polvi NH (1999) The prediction of violence in pre-trial forensic patients: The relative efficacy of statistical versus clinical predictions of dangerousness. Burnaby, BC: Simon Fraser University.
59. Public Safety and Solicitor General: Spousal Assault Risk Assessment (SARA) and Community Risk/Needs Assessment (CRNA): Predictive efficacy and interrelationships. Victoria, BC: Public Safety and Solicitor General, 2004.
60. Ramirez MP, Illescas SR, Garcia MM, Forero CG, Pueyo AA (2008) Prediccion de riesgo de reincidencia en agresores sexuales. [Predicting risk of recidivism in sexual offenders]. Psicothema 20: 205-210.
61. Reeves KA, Kropp PR, Cairns K (2008) An independent validation study of the SARA. Vienna: International Association of Forensic Mental Health Services.
62. Rennie CE, Dolan MC (2010) The significance of protective factors in the assessment of risk. Crim Behav Ment Health 20: 8-22.
63. Rettenberger M, Eher R (2007) Predicting reoffense in sexual offender subtypes: A prospective validation study of the German version of the Sexual Offender Risk Appraisal Guide (SORAG). Sex Offend Treat 2: 1-12.
64. Rettenberger M, Matthes A, Boer DP, Eher R (2010) Prospective actuarial risk assessment: A comparison of five risk assessment instruments in different sexual offender subtypes. Int J Offender Ther Comp Criminol 54: 169-186.
65. Rice ME, Harris GT (2002) Men who molest their sexually immature daughters: Is a special examination required? J Abnorm Psychol 111: 329-339.
66. Serin RC, Mailloux DL, Malcolm PB (2001) Psychopathy, deviant sexual arousal, and recidivism among sexual offenders. J Interpers Viol 16: 234-246.
67. Seto MC, Barbaree HE (1999) Psychopathy, treatment behavior, and sex offender recidivism. J Interpers Viol 14: 1235-1248.
68. Simourd D (2006) Validation of risk/needs assessments in the Pennsylvania Department of Corrections. Lower Allen, PA: Pennsylvania Department of Corrections.
69. Sjöstedt G, Långström N (2001) Actuarial assessment of sex offender recidivism risk: A cross-validation of the RRASOR and the Static-99 in Sweden. Law Hum Behav 25: 629-645.
70. Sjöstedt G, Långström N (2002) Assessment of risk for criminal recidivism among rapists: A comparison of four different measures. Psychol Crim Law 8: 25-40.
71. Snowden RJ, Gray NS, Taylor J, MacCulloch MJ (2007) Actuarial prediction of violent recidivism in mentally disordered offenders. Psychol Med 37: 1539-1549.
72. Snowden RJ, Gray NS, Taylor J, Fitzgerald S (2009) Assessing risk of future violence among forensic psychiatric inpatients with the Classification of Violence Risk (COVR). Psychiatr Serv 60: 1522-1526.
73. Soothill K, Harman J, Francis B, Kirby S (2005) Identifying future repeat danger from sexual offenders against children: A focus on those convicted and those strongly suspected of such crime. J Forensic Psychiatr Psychol 16: 225-247.
74. Sreenivasan S, Garrick T, Norris R, Cusworth-Walker S, Weinberger LE, et al. (2007) Predicting the likelihood of future sexual recidivism: Pilot study findings from a California sex offender risk project and cross-validation of the Static-99. J Am Acad Psychiatr Law 35: 454-468.
75. Stadtland C, Hollweg M, Kleindienst N, Dietl J, Reich U, et al. (2006) Rueckfallprognosen bei Sexualstraftaetern: Vergleich der praediktiven Validitaet von Prognoseinstrumenten. [Predictions of recidivism in sexual offenders: Comparison of the predictive validity of assessment tools]. Nervenarzt 77: 587-595.
76. Thornton D (2002) Constructing and testing a framework for dynamic risk assessment. Sex Abuse 14: 139-153.
77. Viljoen JL, Scalora M, Cuadra L, Bader S, Chavez V, et al. (2008) Assessing risk for violence in adolescents who have sexually offended: A comparison of the J-SOAP-II, J-SORRAT-II, and SAVRY. Crim Justice Behav 35: 5-23.
78. Walkington Z, O’Keeffe C, Thomas S (2006) Predicting violence recidivism in violent juveniles: A UK trial. London, HM Prison Service.
79. Walters G, Duncan S, Geyer M (2003) Predicting disciplinary adjustment in inmates undergoing forensic evaluation: A direct comparison of the PCL-R and the PAI. J Forensic Psychiatr Psychol 14: 382-393.
80. Walters G, Knight RA, Grann M, Dahle KP (2008) Incremental validity of the Psychopathy Checklist facet scores: Predicting release outcome in six samples. J Abnorm Psychol 117: 396-405.
81. Wilcox D, Beech A, Markall HF, Blacker J (2009) Actuarial risk assessment and recidivism in a sample of UK intellectually disabled sexual offenders. Sex Aggress 15: 97-106.
82. Wormith JS, Olver ME, Stevenson HE, Girard L (2007) The long-term prediction of offender recidivism using diagnostic, personality, and risk/need approaches to offender assessment. Psychol Serv 4: 287-305.

## 
